# Assessment of Pretreatment and Posttreatment Evolution of Neurofilament Light Chain Levels in Patients Who Develop Immune Effector Cell–Associated Neurotoxicity Syndrome

**DOI:** 10.1001/jamaoncol.2022.3738

**Published:** 2022-09-01

**Authors:** Omar H. Butt, Alice Y. Zhou, Paolo F. Caimi, Patrick H. Luckett, Julie K. Wisch, Paul-Robert Derenoncourt, Kenneth Lee, Gregory F. Wu, Marcos J. G. de Lima, Jian L. Campian, Matthew J. Frank, John F. DiPersio, Armin Ghobadi, Beau M. Ances

**Affiliations:** 1 Siteman Cancer Center, Department of Medicine, Division of Oncology, Washington University in St Louis, St Louis, Missouri; 2 Department of Hematology and Medical Oncology, Cleveland Clinic, Cleveland Ohio; 3 Department of Neurology, Washington University in St Louis, St Louis, Missouri; 4 Mallinckrodt Institute of Radiology, Division of Nuclear Medicine, Washington University in St Louis, St Louis, Missouri; 5 Department of Hematology, Ohio State University Cancer Treatment and Research Center, The Ohio State University, Columbus; 6 Division of Blood and Marrow Transplantation and Cellular Therapy, Department of Medicine, Stanford University, Stanford, California

## Abstract

**Importance:**

Determining whether neurofilament light chain (NfL) elevations in patients who develop immune effector cell–associated neurotoxicity syndrome (ICANS) occur before or after infusion of cellular product is important to identify high-risk patients and inform whether neuroaxonal injury is latent or a consequence of treatment.

**Objective:**

To quantify serial NfL levels in patients undergoing cellular therapy.

**Design, Setting, and Participants:**

This retrospective 2-center study examined plasma NfL levels in 30 patients with detailed medical and treatment history, including all major pretreatment and posttreatment risk factors. Exclusion criteria included dementia and severe, symptomatic central nervous system (CNS) involvement.

**Main Outcomes and Measures:**

Patients’ NfL levels were measured at 7 time points: baseline (prelymphodepletion), during lymphodepletion, postinfusion day (D) 1, D3, D7, D14, and D30. Prediction accuracy for the development of ICANS was next modeled using receiver operating characteristic (ROC) classification. Finally, univariate and multivariate modeling examined the association between NfL levels, ICANS, and potential risk factors including demographic (age, sex), oncologic (tumor burden, history of CNS involvement), neurologic (history of nononcologic CNS disease or neuropathy), and neurotoxic exposure histories (vincristine, cytarabine, methotrexate, or CNS radiotherapy).

**Results:**

A total of 30 patients (median [range] age, 64 [22-80] years; 12 women [40%] and 18 men [60%]) were included. Individuals who developed ICANS had elevations in NfL prior to lymphodepletion and chimeric antigen receptor T-cell infusion compared with those who did not develop ICANS (no ICANS: 29.4 pg/mL, vs any ICANS: 87.6 pg/mL; *P* < .001). Baseline NfL levels further predicted ICANS development with high accuracy (area under the ROC curve, 0.96), sensitivity (0.91), and specificity (0.95). Levels of NfL remained elevated across all time points, up to 30 days postinfusion. Baseline NfL levels correlated with ICANS severity but not demographic factors, oncologic history, nononcologic neurologic history, or history of exposure to neurotoxic therapies.

**Conclusions and Relevance:**

In a subset of patients in this cross-sectional study, the risk of developing ICANS was associated with preexisting neuroaxonal injury that was quantifiable with plasma NfL level. This latent neuroaxonal injury was present prior to drug administration but was not associated with historic neurotoxic therapies or nononcologic neurologic disease. Preinfusion NfL may further permit early screening and identification of patients most at risk for ICANS. Additional studies are needed to determine NfL’s utility as a predictive biomarker for early (preemptive or prophylactic) intervention and to delineate the origin of this underlying neural injury.

## Key Points

**Question:**

How early are elevations in plasma neurofilament light chain (NfL) levels observed before the development of neurotoxicity after chimeric antigen receptor T-cell therapy?

**Findings:**

In this cross-sectional study of 30 patients undergoing cellular therapy, NfL levels were elevated in patients who developed immune effector cell–associated neurotoxicity syndrome (ICANS) at baseline (preinfusion) and remained elevated for 30 days.

**Meaning:**

Preinfusion NfL elevations suggest ICANS may unmask preexisting neurologic injury present prior to infusion, suggesting that early screening is feasible before drug administration and that inclusion of preexisting neurologic injury may be considered in ICANS pathophysiology models traditionally focusing on the interaction of inflammation and endothelial dysfunction.

## Teaser

This cross-sectional study quantifies serial neurofilament light chain levels in patients undergoing cellular therapy.

## Introduction

After chimeric antigen receptor (CAR) T-cell therapy, 40% to 60% of patients will develop neurotoxicity termed *immune effector cell–associated neurotoxicity syndrome (ICANS)*.^1,2,3^ While the number of patients treated with cellular therapy (<10 000 annually) represents a small fraction of all patients treated for cancer, the indications for cellular therapy are rapidly growing. Symptom onset is typically 3 to 9 days postinfusion and range from encephalopathy to aphasia to cerebral edema.^2,3^ While most cases of low-grade (grade 1-2) ICANS are self-limited, grade 3 or higher ICANS can cause substantial morbidity and mortality.^4^ The early identification of patients at risk for ICANS is critical for preemptive management. Recently, neurofilament light chain (NfL), an axonal structural protein with elevated levels in multiple neurodegenerative and neuroinflammatory diseases,^5^ has emerged as a potential biomarker in ICANS.^6,7^ Schoeberl and colleagues^7^ reported postinfusion NfL elevations up to 5 days prior to peak ICANS. However, it remains unclear if this is an acute postinfusion elevation or a chronic change predating cellular therapy. Likewise, NfL’s association with established ICANS risk factors (preinfusion baseline tumor burden, CAR T-cell dose, history of preexisting neurologic comorbidities,^8^ and postinfusion ferritin level, lactate dehydrogenase (LDH) level, platelet count, fibrinogen level, and C-reactive protein [CRP] level^3,8,9,10,11^) is unclear. In this study, we investigate serial plasma NfL levels in patients undergoing cellular therapy, starting from before lymphodepletion to 30 days postinfusion, and examine its association with ICANS and potential risk factors.

## Methods

### Participants

Thirty patients treated with a CD19 CAR T-cell therapy at Washington University in Saint Louis (WU) and Case Western Reserve (CW) were evaluated. Patients at WU were participants from the Center for Gene and Cellular Immunotherapy registry (July 1, 2019, to December 31, 2020), and patients at CW were enrolled in a phase 1 trial with locally manufactured CAR T cells.^12^ Cohort size was limited to all available samples that met inclusion and exclusion criteria (eMethods in the Supplement). The American Society for Transplantation and Cellular Therapy criteria defined ICANS and cytokine release syndrome (CRS) grading.^4^ Each site’s respective institutional review board approved the study, and written consent was obtained as part of the aforementioned study and trial.

### Biomarker Quantification and Clinical Covariates

Neurofilament light chain level was assayed using a Simoa HD-X kit (Quanterix) from archived samples. Time points included baseline (before lymphodepletion), lymphodepletion (preinfusion), day 1 (D1), D3, D7, D14, and D30. Laboratory correlates, including platelet count and CRP, LDH, fibrinogen, and ferritin levels, were quantified in each site’s clinical laboratory for available time points (lymphodepletion, D1, D3, D5, and D7).

### Statistical Analysis

Wilcoxon rank-sum tests evaluated univariate comparisons between ICANS grade 1 or higher vs grade 0, and ICANS grade 3 or higher vs grade 0 to 2. False discovery rate corrected for multiple comparisons. Each biomarker then served as a predictor in a binomial logistic regression model for ICANS status. The regression model probability estimates defined a receiver operating characteristic (ROC) curve. The area under the ROC curve (AUC) and optimal operating point of a given ROC curve then determined model accuracy, with validation on 10 000 random permutations of 24 of the 30 patients.

Point biserial correlation compared NfL level with categorical covariates (sex, history of CNS involvement, nononcologic CNS disease, neuropathy, or vincristine, cytarabine, high-dose methotrexate, intrathecal methotrexate, or CNS radiotherapy exposure). Rank-correlation compared continuous (NfL, CRP, LDH, fibrinogen, and ferritin levels, platelet count, age, tumor burden) and ordinal (ICANS grade, CRS grade) variables. The absolute value of the resulting cross-correlation matrix subserved agglomerative hierarchical clustering. Multivariate modeling included data-driven (Lasso regression) and a priori–defined (partial correlation) approaches to model ICANS grade (the outcome variable; eMethods in the Supplement). All analyses were performed using Matlab, version R2020a (Mathworks).

## Results

### Baseline NfL Levels

We examined baseline (prelymphodepletion) NfL levels in 30 individuals (median [range] age, 64 [22-80] years; 12 women [40%] and 18 men [60%]; 23 [77%] with a history of diffuse large B-cell lymphoma) treated with CD19 CAR T cells (63% axicabtagene ciloleucel; Table 1). Patients who developed any grade ICANS, low-grade (grade 1-2) ICANS, and grade 3 or higher ICANS had significant elevations in baseline NfL level (mean, 87.6 pg/mL, 115.3 pg/mL, and 71.7 pg/mL, respectively) compared with the grade 0 group (29.4 pg/mL; Figure, A). The grade 1 to 2 and grade 3 or higher subgroups did not differ from one another.

**Table 1.** Patient Characteristics^a^

Characteristic	No. (%)		
	All (n = 30)	No ICANS (n = 19)	Any ICANS (n = 11)
Age, median (range), y	64 (22-80)	64 (22-80)	64 (33-79)
Sex			
Female	12 (40)	7 (37)	5 (45)
Male	18 (60)	12 (63)	6 (55)
Race			
Asian	1 (3)	1 (5)	0
Black	2 (7)	1 (5)	1 (18)
Hispanic	1 (3)	1 (5)	0
White	26 (87)	16 (84)	10 (91)
Cancer history			
DLBCL	23 (77)	15 (79)	8 (73)
Stage at initial diagnosis, median	3	3	3
Tumor burden,^b^ mean (SD), mm^3^	133.1 (233)	140.2 (288)	122.1 (117)
CNS involvement	7 (23)	5 (26)	2 (18)
History of			
Vincristine exposure	28 (93)	18 (95)	10 (91)
Cytarabine exposure	6 (20)	3 (16)	3 (2)
High-dose methotrexate	5 (17)	3 (16)	2 (18)
Intrathecal methotrexate	5 (17)	3 (16)	2 (18)
CNS radiotherapy	3 (10)	3 (16)	0
Neurologic history			
Neurologic disease, not related to cancer	5 (17)	2 (10)	3 (27)
Neuropathy	17 (57)	10 (52.6)	7 (63.6)
CAR T-cell product			
Axicabtagene ciloleucel	19 (63)	12 (63)	7 (64)
Tisagenlecleucel	4 (13)	3 (16)	1 (9)
Brexucabtagene autoleucel	1 (3)	0	1 (9)
Experimental CD19 CAR (2nd generation)	6 (20)	4 (21)	2 (18)

Abbreviations: CAR, chimeric antigen receptor; CD19, cluster of differentiation 19; CNS, central nervous system; DLBCL, diffuse large B-cell lymphoma; ICANS, immune effector cell–associated neurotoxicity syndrome.

^a^ Demographic and oncologic characteristics of the study cohort. Expanded version with ICANS subgroups is in the eTable in the Supplement.

^b^ Mean tumor volume was derived from total lesion burden on preinfusion positron emission tomography scans (see eMethods in the Supplement).

**Figure.**
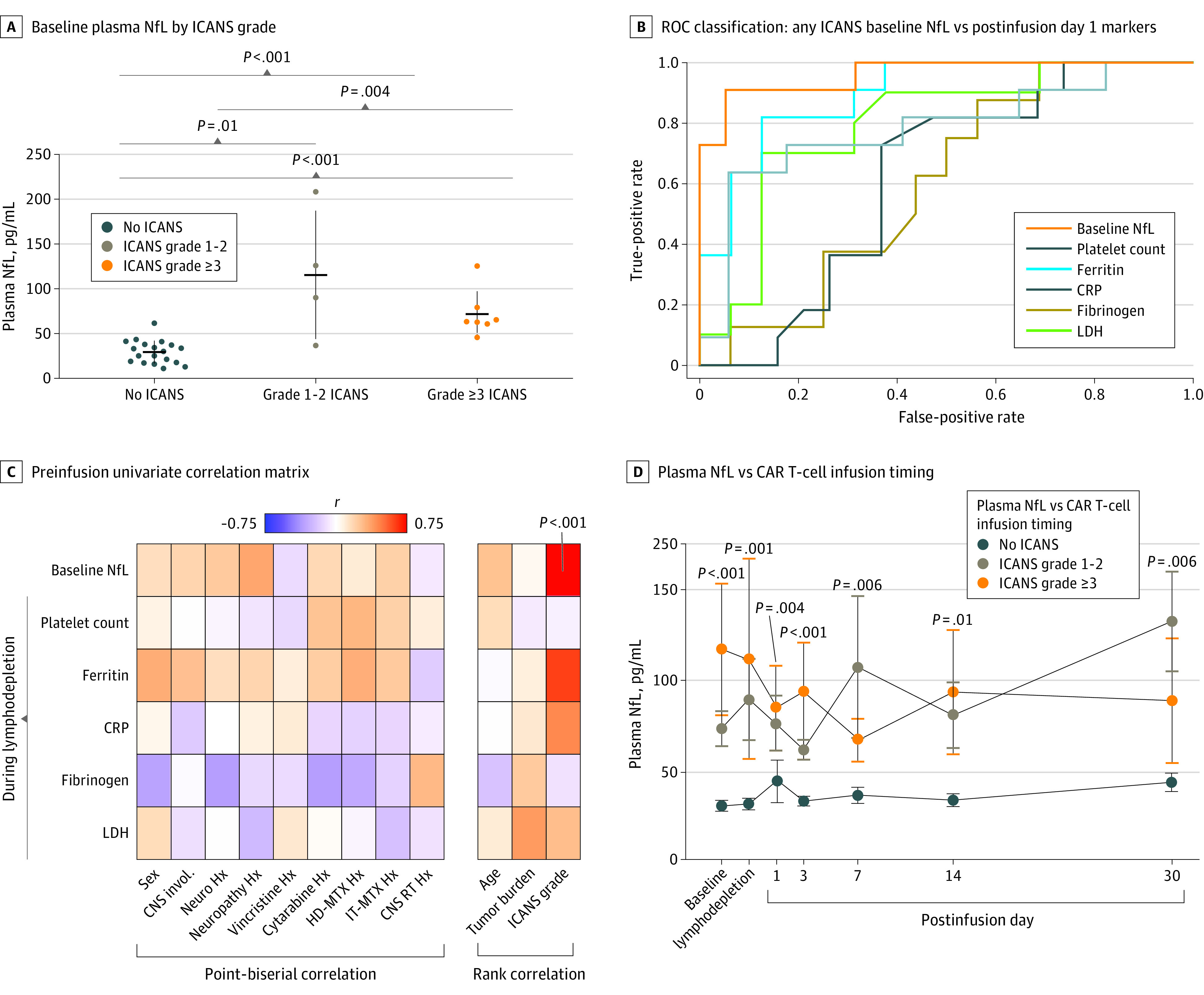
Association Between Neurofilament Light Chain (NfL) Levels and Immune Effector Cell–Associated Neurotoxicity Syndrome A, Baseline (preinfusion) plasma NfL levels divided by immune effector cell–associated neurotoxicity syndrome (ICANS) grade. B, Receiver operating characteristic (ROC) classification of patients who develop any grade ICANS (≥1) vs no ICANS (grade 0) for baseline NfL level and available postinfusion day 1 markers. C, Univariate correlation between pretreatment factors and available preinfusion biomarkers. D, Blood plasma NfL levels relative to timing of lymphodepletion and chimeric antigen receptor (CAR) T-cell infusion; group comparisons here were between any grade ICANS (≥1) vs no ICANS (grade 0). Multiple comparison validation per false discovery rate. CNS indicates central nervous system; CRP, C-reactive protein; HD, high-dose; Hx, history; IT, intrathecal; LDH, lactate dehydrogenase; Neuro, neurologic disease; MTX, methotrexate; RT, radiotherapy.

### Classification Accuracy

Baseline NfL accurately predicted ICANS development (AUC, 0.96; sensitivity, 0.91; specificity, 0.95; Figure, B), surpassing postinfusion markers across all time points (eFigure 1 in the Supplement). Likewise, hierarchical clustering grouped ICANS grade with NfL (eFigures 2 and 3 in the Supplement). Comparable results were observed for NfL between grade 3 or higher ICANS and the combined grade 0 to 2 ICANS cohort (AUC, 0.86; sensitivity, 1.0; specificity, 0.82; eFigures 4 and 5 in the Supplement).

### Association Between Serial NfL Levels, ICANS, and Potential Risk Factors

Baseline NfL level correlated with ICANS grade (*r* = 0.74; *P* < .001). No association was observed with demographic, oncologic, neurologic, or exposure to neurotoxic risk factors, including CRS (Figure, C). Multivariate modeling likewise identified NfL level as the main contributing factor to ICANS grade (eFigure 6 and eResults in the Supplement). Finally, NfL levels remained elevated in individuals who developed ICANS for 30 days after infusion (Figure, D) with persistent correlation with ICANS severity across all time points (eFigure 7 in the Supplement). The classification accuracy of NfL persisted across available time points, despite smaller group sizes (Table 2).

**Table 2.** NfL Classification Accuracy for ICANS Development at Different Time Points^a^

Time point	No.	ICANS, %	AUC	Sensitivity	Specificity
Baseline	30	37	0.96	0.91	0.95
Lymphodepletion	23	35	0.93	0.88	0.93
D1	24	38	0.87	0.89	0.87
D3	24	38	0.96	0.89	0.93
D7	24	38	0.84	0.67	0.93
D14	21	33	0.84	0.50	1.0
D30	21	40	0.88	0.57	1.0

Abbreviations: AUC, area under the ROC curve; ICANS, immune effector cell–associated neurotoxicity syndrome; NfL, neurofilament light chain; ROC, receiver operating characteristic.

^a^ The ROC classification of patients who develop any grade ICANS (≥1) vs no ICANS (grade 0) for NfL at each of the available time points. Baseline refers to prior to lymphodepletion, while lymphodepletion refers to blood draws obtained during lymphodepletion, but prior to chimeric antigen receptor infusion. D refers to the postinfusion day (eg, D1 is postinfusion day 1). Note that only a subset of samples were available for the later time points.

## Discussion

ICANS remains an enigmatic neurologic syndrome whose pathophysiology remains poorly understood. A need remains to identify patients most at risk prior to onset given the high morbidity/mortality and potential for rapid clinical decline. Here, we show that preinfusion plasma NfL levels were a robust marker for ICANS development. The association between NfL level and ICANS grade persisted independent of potential confounds, including age, sex, tumor burden, history of neurologic disease, and history of neurotoxic therapies. Plasma NfL levels also remained elevated after infusion for up to 30 days after infusion.

Critically, this study demonstrates that NfL elevations are present prior to drug administration in a cohort of patients treated with CD19-directed cellular therapy. Preliminary work suggested NfL elevations may be observed 5 days prior to peak ICANS,^7^ but it was unknown if this reflected an acute change after drug infusion or chronic elevations that predate lymphodepletion and infusion.

While unlikely to be the sole driver of ICANS, neural injury reflected by NfL may aid in identifying a high-risk subset of patients undergoing cellular therapy. However, NfL’s association with current models of ICANS pathophysiology remains unclear. Current models center on systemic inflammatory changes leading to endothelial dysfunction, blood-brain barrier breakdown,^8^ and systemic cytokine and/or monocytes infiltration into the CNS, resulting in neuroinflammation and symptoms.^8,9,11^ These models underappreciate predisposing risk factors for ICANS development, such as neurologic injury.^8^ Whether this injury reflects a subclinical neuroinflammatory process or a microvascular process affecting axon health remains unclear.^13^ Given that known endothelial and blood-brain barrier dysfunction is associated with ICANS,^8^ the latter is more plausible.

The source of injury remains unknown, with no clear association with exposure to neurotoxic therapy, though this may reflect low power. ICANS has been associated with acute white matter (WM) microvascular and macrovascular injury on imaging, including hemorrhagic and nonhemorrhagic encephalitis and strokes.^14^ Incidentally, NfL elevations are also associated with WM lesions and neuroinflammatory and vascular insults such as stroke, encephalitis, and lesion burden in multiple sclerosis.^5,13,15^ Together this suggests possible subclinical WM injury through microvascular injury via an as of yet uncharacterized pathway. Persistent 30-day postinfusion elevations suggest that while the acute, symptomatic phase of ICANS may resolve, persistent occult neurologic injury persists. Finally, correlation with a systemic process like CRS would be unusual given NfL’s neuroaxonal origin. Indeed, no correlation was observed between NfL level and CRS in the current cohort, despite significant association between CRS and ICANS (*r* = 0.49; *P* = .006; eResults in the Supplement).

### Limitations

Study limitations include a cohort treated predominantly with axicabtagene ciloleucel, insufficient power for subgroup comparisons between ICANS grades, and a lack of available cerebrospinal fluid samples or imaging to provide additional information about the underlying pathophysiology.

## Conclusions

In this cross-sectional study, the risk of developing ICANS was associated with preexisting neuroaxonal injury that was quantifiable with plasma NfL level in a subset of patients. This latent neuroaxonal injury was present prior to drug administration but was not associated with historic neurotoxic therapies or nononcologic neurologic disease. Additional studies are needed to examine preinfusion NfL elevations as a biomarker for prophylaxis or early intervention in patients at risk for ICANS.

## Notes

Supplement.
eMethods

eResults
**eTable.** Demographic and oncologic characteristics of the study cohort **eFigure 1.** Receiver operating characteristic curve classification of patients who develop any grade ICANS (1+) vs grade 0 for Platelet Count, Ferritin, CRP, Fibrinogen, LDH **eFigure 2.** Univariate rank (Spearman’s) cross-correlation matrix comparing post-infusion day 1 (D1) blood biomarkers to baseline NfL, age, and total tumor burden and hierarchical clustering of the same terms **eFigure 3.** Univariate rank (Spearman’s) cross-correlation matrix comparing during lymphodepletion blood biomarkers to baseline NfL, age, and total tumor burden and hierarchical clustering of the same terms **eFigure 4.** Receiver operating characteristic curve classification of patients who develop grade 3+ ICANS vs grade 0-2 ICANS for baseline NfL and available post-infusion day 1 (D1) markers **eFigure 5.** Receiver operating characteristic curve classification of patients who develop grade 3+ ICANS vs grade 0-2 for Platelet Count, Ferritin, CRP, Fibrinogen, and LDH **eFigure 6.** Cross-Validation (12x) Mean Squared Error plot reflecting lasso (elastic) regression of 12 pre-treatment factors **eFigure 7.** Univariate correlation between ICANS grade and available biomarkers at different time-points relative to lymphodepletion and CAR T infusion
