# Supplementary material for: Assessment of Pretreatment and Posttreatment Evolution of Neurofilament Light Chain Levels in Patients Who Develop Immune Effector Cell–Associated Neurotoxicity Syndrome
Source: JAMA Oncol. 2022 Sep 1;8(11):1652–7. doi: 10.1001/jamaoncol.2022.3738 (PMC9437827; doi:10.1001/jamaoncol.2022.3738)
Supplement: Supplement. — eMethods eResults eTable. Demographic and oncologic characteristics of the study cohort eFigure 1. Receiver operating characteristic curve classification of patients who develop any grade ICANS (1+) vs grade 0 for Platelet Count, Ferritin, CRP, Fibrinogen, LDH eFigure 2. Univariate rank (Spearman’s) cross-correlation matrix comparing post-infusion day 1 (D1) blood biomarkers to baseline NfL, age, and total tumor burden and hierarchical clustering of the same terms eFigure 3. Univariate rank (Spearman’s) cross-correlation matrix comparing during lymphodepletion blood biomarkers to baseline NfL, age, and total tumor burden and hierarchical clustering of the same terms eFigure 4. Receiver operating characteristic curve classification of patients who develop grade 3+ ICANS vs grade 0-2 ICANS for baseline NfL and available post-infusion day 1 (D1) markers eFigure 5. Receiver operating characteristic curve classification of patients who develop grade 3+ ICANS vs grade 0-2 for Platelet Count, Ferritin, CRP, Fibrinogen, and LDH eFigure 6. Cross-Validation (12x) Mean Squared Error plot reflecting lasso (elastic) regression of 12 pre-treatment factors eFigure 7. Univariate correlation between ICANS grade and available biomarkers at different time-points relative to lymphodepletion and CAR T infusion [file jamaoncol-e223738-s001.pdf]

Supplemental Online Content

Butt OH, Zhou AY, Caimi PF, et al. Assessment of pretreatment and posttreatment evolution of neurofilament light chain levels in patients who develop immune effector cell–associated neurotoxicity syndrome. *JAMA Oncol.* Published online September 1, 2022. doi:10.1001/jamaoncol.2022.3738

eMethods

eResults

eTable. Demographic and oncologic characteristics of the study cohort

eFigure 1. Receiver operating characteristic curve classification of patients who develop any grade ICANS (1+) vs grade 0 for Platelet Count, Ferritin, CRP, Fibrinogen, LDH

eFigure 2. Univariate rank (Spearman's) cross-correlation matrix comparing post-infusion day 1 (D1) blood biomarkers to baseline NfL, age, and total tumor burden and hierarchical clustering of the same terms

eFigure 3. Univariate rank (Spearman's) cross-correlation matrix comparing during lymphodepletion blood biomarkers to baseline NfL, age, and total tumor burden and hierarchical clustering of the same terms

eFigure 4. Receiver operating characteristic curve classification of patients who develop grade 3+ ICANS vs grade 0-2 ICANS for baseline NfL and available post-infusion day 1 (D1) markers

eFigure 5. Receiver operating characteristic curve classification of patients who develop grade 3+ ICANS vs grade 0-2 for Platelet Count, Ferritin, CRP, Fibrinogen, and LDH

eFigure 6. Cross-Validation (12x) Mean Squared Error plot reflecting lasso (elastic) regression of 12 pre-treatment factors

eFigure 7. Univariate correlation between ICANS grade and available biomarkers at different time-points relative to lymphodepletion and CAR T infusion

This supplemental material has been provided by the authors to give readers additional information about their work.

© 2022 Butt OH et al. *JAMA Oncology.*

A. Supplemental Methods

Expanded Variable definitions

Pre-Infusion, Pre-lymphodepletion Variables

Name	Variable Type	Origin
Sex	Categorical	Demographics
CNS Involvement	Categorical	Pre-infusion medical history
History of Neurologic Disease*	Categorical	Pre-infusion medical history
History of Neuropathy	Categorical	Pre-infusion medical history
History of Vincristine	Categorical	Pre-infusion medical history
History of Cytarabine	Categorical	Pre-infusion medical history
History of HD Methotrexate	Categorical	Pre-infusion medical history
History of IT Methotrexate	Categorical	Pre-infusion medical history
History of CNS Radiotherapy	Categorical	Pre-infusion medical history
Tumor Burden	Continuous	Per Pre-infusion PET Scan**
Plasma NfL	Continuous	Blood Draw prior to infusion***

***** Two patients had a history of stroke, two patients had a history of migraines, and one patient had a history of seizures. One patient with a history of stroke developed Grade 1 ICANS while the other developed Grade 4 ICANS. Neither patient with a history of migraine developed ICANS. Finally, the patient with a history of seizures developed Grade 1 ICANS.

******Tumor burden was derived from the most recent PET scan prior to CAR T infusion (median 44 days (range [4-94]) for the complete cohort; median 45.5 days (range [7-94]) for the no ICANS group, median 34 days (range [4-81]) for ICANS group). Tumor burden was defined as the mean metabolic tumor volume (MTV) for each patient. First, ellipsoidal regions of interest (ROIs) were defined on attenuation-corrected images using the Affinity Viewer of the Hermes software package (Hermes Medical Solutions, Stockholm, Sweden). ROIs were next manually adjusted not to include adjacent physiologic activity. A local threshold of 41% of the maximum SUV for each defined lesion was used for segmentation (1,2). When true sites of disease were not confidently distinguished from physiological uptake, only focal lesions were included. The sum of the MTVs of all identifiable hypermetabolic lesions defined the patient MTV.

***Baseline plasma NfL was obtained from archived (single-freeze/thaw) plasma obtained prior to CAR T infusion (median 9.5 days (range [3-61]) for the complete cohort; median 25 days (range [4-61]) for the no ICANS group, median 7 days (range [3-26]) for ICANS group).

Pre-Infusion, Post-lymphodepletion Variables

© 2022 Butt OH et al. *JAMA Oncology.*

Platelet Count	Continuous	Earliest available laboratory data during lymphodepletion
Ferritin	Continuous	Earliest available laboratory data during lymphodepletion
LDH	Continuous	Earliest available laboratory data during lymphodepletion
CRP	Continuous	Earliest available laboratory data during lymphodepletion
Fibrinogen	Continuous	Earliest available laboratory data during lymphodepletion
Plasma NfL	Continuous	Archived plasma obtained during lymphodepletion, after the baseline sample and prior to infusion

Post-Infusion Variables

Platelet Count	Continuous	Laboratory data on D1, D3, D5, and D7
Ferritin	Continuous	Laboratory data on D1, D3, D5, and D7
LDH	Continuous	Laboratory data on D1, D3, D5, and D7
CRP	Continuous	Laboratory data on D1, D3, D5, and D7
Fibrinogen	Continuous	Laboratory data on D1, D3, D5, and D7
Plasma NfL	Continuous	Archived plasma obtained on D1, D3, D7, D14, and D30
CRS Grade	Ordinal	Peak CRS Grade
ICANS Grade	Ordinal	Peak ICANS Grade

Cohort Size

Cohort size was determined by extrapolating from preliminary work where baseline NfL for patients undergoing cellular therapy who did not develop ICANS (n = 9) was 28.3 +/- 9.1SD (Butt, et al, 2020). NfL levels in patients who developed ICANS was significantly higher (45.6 pg/ml, 125.2 pg/ml, and 62.8 pg/ml). Based on a conservative estimate of a 20% increase with an assumed 80% power and an alpha of 0.05, a target study group of 20 patients was needed assuming 10 ICANS patients and 10 non-ICANS patients. Thus, a target goal of at least 10 evaluable ICANS patients determined the inclusion window of 1.5 years. Inclusion criteria included available pre-infusion plasma (up to 6 weeks prior to completion of lymphodepletion). Exclusion criteria included dementia (n=1) or severe central nervous system (CNS) involvement (n=1). Exclusion criteria was defined to minimize secondary potential sources of NfL elevation which may bias the results (Khalil et al, 2018)

© 2022 Butt OH et al. *JAMA Oncology.*

B. Supplemental Results

Detailed Patient Demographics

ID	Age	Sex	Race	Cancer History	CAR T product	Peak ICANS Score	Peak CRS Score
1	75	M	white	FL->DLBCL	Axicabtagene Ciloleucel	3	2
2	60	M	asian	DLBCL	Axicabtagene Ciloleucel	0	2
3	58	M	white	DLBCL	Axicabtagene Ciloleucel	0	1
4	70	M	white	DLBCL	Axicabtagene Ciloleucel	0	1
5	58	F	black	DLBCL/PTLD	Axicabtagene Ciloleucel	3	1
6	67	M	white	DLBCL	Tisagenlecleucel	0	0
7	60	M	white	DLBCL	Axicabtagene Ciloleucel	3	2
8	67	F	white	DLBCL	Axicabtagene Ciloleucel	0	0
9	81	M	white	PCBCL->DLBCL	Axicabtagene Ciloleucel	0	0
10	77	M	white	MZL->DLBCL	Axicabtagene Ciloleucel	0	1
11	22	M	white	pre-B ALL	Tisagenlecleucel	0	1
12	64	M	white	FL-->DLBCL	Tisagenlecleucel	0	1
13	79	M	white	DLBCL	Tisagenlecleucel	4	1
14	65	M	white	DLBCL	Axicabtagene Ciloleucel	0	1
15	78	F	white	FL->DLBCL	Axicabtagene Ciloleucel	1	1
16	68	F	white	FL-->DLBCL	Axicabtagene Ciloleucel	0	2
17	57	M	black	DLBCL/PTLD	Axicabtagene Ciloleucel	0	1
18	57	F	white	PMBL	Axicabtagene Ciloleucel	0	2
19	69	F	white	DLBCL	Axicabtagene Ciloleucel	3	2
20	58	M	white	DLBCL	Axicabtagene Ciloleucel	1	2
21	71	F	white	DLBCL/FL	Axicabtagene Ciloleucel	0	0
22	61	F	white	DLBCL	Axicabtagene Ciloleucel	1	0
23	59	F	white	DLBCL/FL	Axicabtagene Ciloleucel	0	1
24	79	M	white	Mantle Cell/MIPI 6.9	Brexucabtagene Autoleuce	4	2
25	66	F	white	T-FL	Experimental	0	1
26	33	M	white	BL	Experimental	4	2
27	45	M	white	DLBCL	Experimental	0	0
28	64	F	white	T-LPL	Experimental	2	2
29	47	F	Hispanic	T-FL	Experimental	0	0
30	60	M	white	DLBCL	Experimental	0	1

Core Demographic and oncologic characteristics of the study cohort, by participant ID. For the patients who developed CRS, there was a median onset of 1 day after infusion (range 0 to 9

© 2022 Butt OH et al. *JAMA Oncology.*

days). For the patients who developed ICANS, there was a median onset of 6 days (range 1 to 16). The experimental CAR product was a second generation CAR targeting CD19.

Permutation testing: NfL vs Ferritin (during lymphodepletion and D1) Classification Accuracy for ICANS

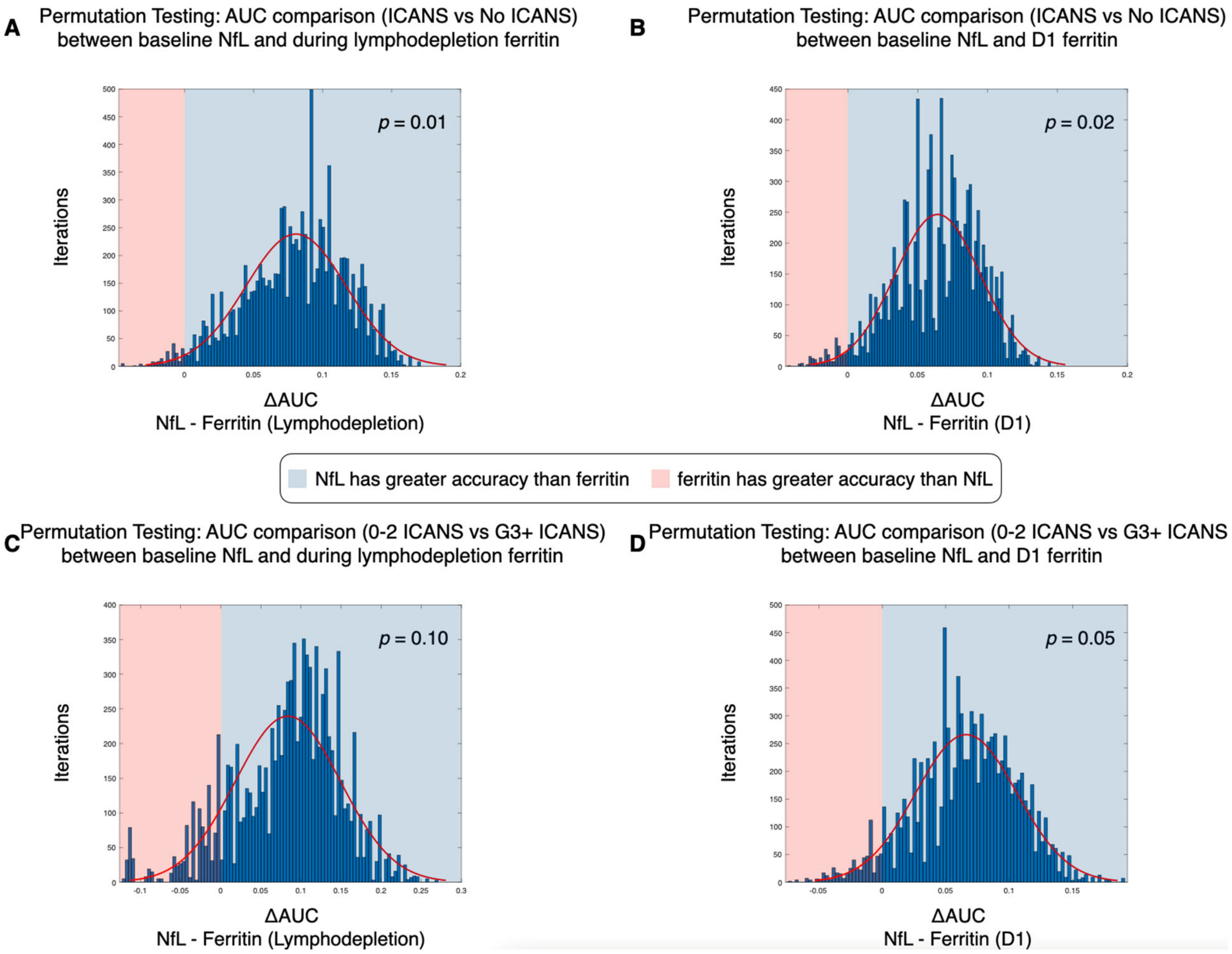

Histograms of the 10,000 permutations on 80% subsets of the data (i.e. 24 of 30 patients) comparing the difference in the AUC between NfL and ferritin for the development of ICANS (**A**, **B**) and for grade 3+ ICANS (**C**,**D**). Both NfL and ferritin had the highest AUC of available markers. An AUC difference greater than zero (blue) implies NfL has a greater AUC. An AUC difference less than zero (red) implies ferritin has a greater AUC.

© 2022 Butt OH et al. *JAMA Oncology.*

Multivariate Lasso Regression: During Lymphodepletion Markers

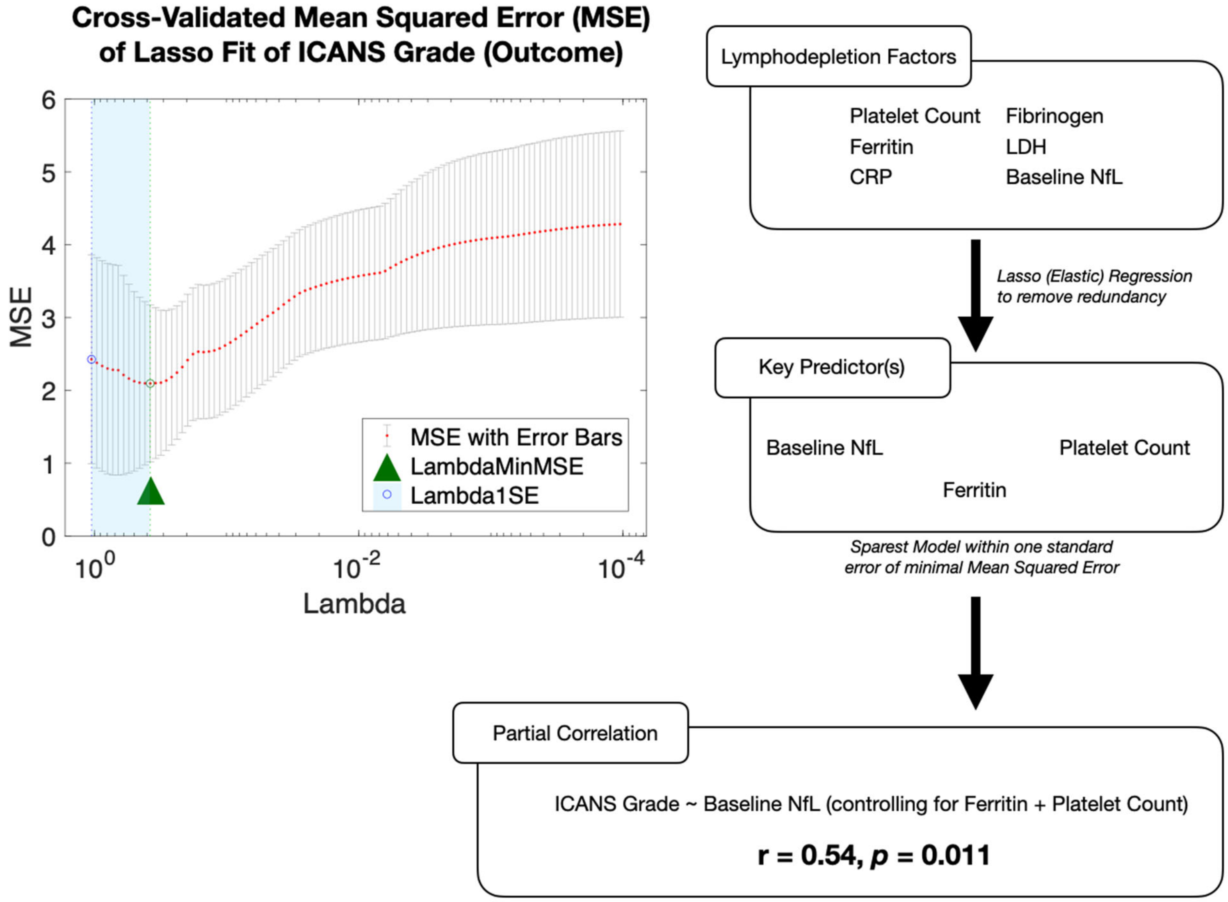

Cross-Validation Mean Squared Error plot reflecting Lasso (elastic) regression for lymphodepletion laboratory data and baseline NfL. Earlier reported Lasso regression (**Supplemental Fig 7**) included only all pre-treatment (i.e. pre-Infusion, pre-lymphodepletion) variables. It did not include data during lymphodepletion. Here Lasso regression was repeated using available lymphodepletion laboratory values and baseline NfL. Three predictors of interest were identified (NfL, Ferritin, and Platelet Count). Subsequent partial correlation examined the relationship between baseline NfL and ICANS grade, after accounting for ferritin and platelet count.

Multivariate Lasso Regression: D1 Markers Markers

Comparable results to above were obtained using D1 laboratory data instead of day of during lymphodepletion data: Again Baseline NFL, D1 ferritin, and D1 platelet count were identified using Lasso regression. After accounting for D1 ferritin and platelet count, baseline NfL remained correlated to ICANS grade (r = 0.58, p = 0.003).

© 2022 Butt OH et al. *JAMA Oncology.*

Correlation between CRS and ICANS

Both CRS and ICANS are ordinal variables, warranting rank (Spearman) correlation evaluation. Rank correlation between ICANS grade and CRS grade showed a significant relationship (**r = 0.49, *p* = 0.0056**). This is likely an underestimate as patients who developed CRS were all treated. Nonetheless, CRS is an important confound warranting further investigation using *a priori* defined models.

A priori-defined Partial Correlations between Baseline NfL and ICANS Grade

Lasso regression and hierarchical clustering are data driven approaches to identify risk factors (or groups of risk factors) that are most related to ICANS grade. An alternative approach is the *a priori* definition of potential risk factors based on previously published data, such as CRS as outlined above. Partial correlations examine the relationship between baseline NFL and ICANS grade after accounting for these pre-defined risk factors. Note, ICANS grade is an ordinal, not continuous variable, hence the use of rank (Spearman) correlations over traditional linear models.

ICANS ~ Baseline NFL (r = 0.74, p = 0.000003)	
Pre-Infusion	- Controlling for Age (r = 0.73, p = 0.000006)
	- Controlling for Age, Tumor Burden (r = 0.73, p = 0.0002)
	- Controlling for Age, Tumor Burden, Neuro Disease (r = 0.72, p = 0.0004)
ALL:	- Controlling for Age, Tumor Burden, Neuro Disease, CRS, Ferritin, Plt, LDH, Fib (r = 0.59, p = 0.04)
Post-Infusion	- Controlling for CRS, Ferritin, Plt, LDH, Fib (r = 0.64, p = 0.004)
	- Controlling for CRS, Ferritin, Plt, LDH (r = 0.51, p = 0.02)
	- Controlling for CRS, Ferritin, Plt (r = 0.58, p = 0.003)
	- Controlling for CRS, Ferritin (r = 0.56, p = 0.004)
	- Controlling for CRS (r = 0.73, p = 0.000006)

Neuro Disease = History of Neurologic disease other than neuropathy
CRS = History of Cytokine Release Syndrome
Plt = Platelet Count
LDH = Lactate Dehydrogenase
Fib = Fibrinogen

Subgroup Analysis: Axi-cel

Subgroup analysis of NfL levels in our 19 patients treated with Axi-cel demonstrate excellent AUC for the development of any grade ICANS (0.95) and G3+ ICANS (0.85), potentially serving as a source of bias.

© 2022 Butt OH et al. *JAMA Oncology.*

Serial Changes in NfL Across Time-points

There was significant group differences between patients with and without ICANS at lymphodepletion ($p = 0.0016$), D3 ($p = 0.004$), D7 ($p = 0.0188$), D14 ($p = 0.003$), and D30 ($p = 0.0011$). All p values survived FDR correction for multiple comparisons. A trend was observed at D1 ($p = 0.095$) (**Figure 1G**). However, there was no significant change from baseline to post-infusion in patients who developed ICANS. This may be a reflection of power.

REFERENCES

1. Butt O, Zhou A, Schindler S, Fagan A, Dipersio J, Ghobadi A, Ances B. NCMP-24. PRE-INFUSION NEUROFILAMENT LIGHT CHAIN (NFL) LEVELS PREDICT THE DEVELOPMENT OF IMMUNE EFFECTOR CELL-ASSOCIATED NEUROTOXICITY SYNDROME (ICANS). Neuro Oncol. 2020 Nov 9;22(Suppl 2):ii127–8. doi: 10.1093/neuonc/noaa215.535. PMCID: PMC7651371.
2. Dean EA, Mhaskar RS, Lu H, Mousa MS, Krivenko GS, Lazaryan A, Bachmeier CA, Chavez JC, Nishihori T, Davila ML, Khimani F, Liu HD, Pinilla-Ibarz J, Shah BD, Jain MD, Balagurunathan Y, Locke FL. High metabolic tumor volume is associated with decreased efficacy of axicabtagene ciloleucel in large B-cell lymphoma. Blood Adv. 2020 Jul 28;4(14):3268-3276. doi: 10.1182/bloodadvances.2020001900. PMID: 32702097; PMCID: PMC7391155.
3. Khalil M, Teunissen CE, Otto M, Piehl F, Sormani MP, Gattringer T, Barro C, Kappos L, Comabella M, Fazekas F, Petzold A, Blennow K, Zetterberg H, Kuhle J. Neurofilaments as biomarkers in neurological disorders. Nat Rev Neurol. 2018 Oct;14(10):577-589. doi: 10.1038/s41582-018-0058-z. PMID: 30171200.
4. Sasanelli M, Meignan M, Haioun C, Berriolo-Riedinger A, Casasnovas RO, Biggi A, Gallamini A, Siegel BA, Cashen AF, Véra P, Tilly H, Versari A, Itti E. Pretherapy metabolic tumour volume is an independent predictor of outcome in patients with diffuse large B-cell lymphoma. Eur J Nucl Med Mol Imaging. 2014

© 2022 Butt OH et al. *JAMA Oncology.*

C. Supplemental Table. Expanded Patient Characteristics

	All (n = 30)	No ICANS (n = 19)	G1-2 ICANS (n = 4)	G3+ ICANS (n=7)
Age, median (range)	64 [22-80]	64 [22-80]	62.5 [58-78]	69 [33-79]
Sex (n (%) Female)	12 (40.0%)	7 (36.8%)	3 (75%)	2 (28.6%
Race (n (%))				
Caucasian	26 (86.7%)	16 (84.2%)	4 (100%)	6 (85.7%)
Other	4 (13.3%)	3 (15.8%)	0 (0%)	1 (14.3%)
Cancer History				
n (%) DLBCL	23 (76.7%)	15 (78.9%)	3 (75%)	5 (71.4%)
Median Stage at Initial Diagnosis	3	3	3	4
Tumor Burden*, mm^3 (mean (std))	133.1 (233)	140.2 (288)	61.47 (51.8)	152.5 (133)
CNS Involvement (n (%))	7 (23.3%)	5 (26.3%)	2 (50%)	0 (0%)
Hx of Vincristine Exposure (n (%))	28 (93.3%)	18 (94.7%)	4 (100%)	6 (85.7%)
Hx of Cytabrine Exposure (n (%))	6 (20%)	3 (15.8%)	1 (25%)	2 (28.6%)
Hx of High-dose Methotrexate (n (%))	5 (16.7%)	3 (15.8%)	1 (25%)	1 (14.3%)
Hx of Intrathecal Methotrexate (n (%))	5 (16.7%)	3 (15.8%)	1 (25%)	1 (14.3%)
Hx of CNS Radiotherapy (n (%))	3 (10%)	3 (15.8%)	0 (0%)	0 (0%)
Neurologic History				
Hx of Neurologic disease, not relating to cancer (n (%))	5 (16.7%)	2 (10.5%)	2 (50%)	1 (14.3%)
Hx of Neuropathy (n (%))	17 (56.7%)	10 (52.6%)	3 (75%)	4 (57.1%)
CAR T Product				
Axicabtagene Ciloleucel (n (%))	19 (63.3%)	12 (63.2%)	3 (75%)	4 (57.1%)
Tisagenlecleucel (n (%))	4 (13.3%)	3 (15.8%)	0 (0%)	1 (14.3%)
Brexucabtagene Autoleucel (n (%))	1 (3.33%)	0 (0%)	0 (0%)	1 (14.3%)
Experimental CD19 CAR (n (%))	6 (20%)	4 (21.1%)	1 (25%)	1 (14.3%)

eTable. Demographic and oncologic characteristics of the study cohort, with the ICANS cohort subdivided into low grade (G1-2) and high grade (G3+). *Mean tumor volume was derived from total lesion burden on pre-infusion positron emission tomography (PET) scans using a 41% maximum standard uptake value (SUV) threshold (1,2); DLBL - Diffuse large B-cell lymphoma; CAR - Chimeric antigen receptor; CD19 - Cluster of Differentiation 19; CNS – Central nervous system; ICANS - Immune effector cell-associated neurotoxicity syndrome; Hx – History; std – standard deviation

1. Dean EA, Mhaskar RS, Lu H, Mousa MS, Krivenko GS, Lazaryan A, Bachmeier CA, Chavez JC, Nishihori T, Davila ML, Khimani F, Liu HD, Pinilla-Ibarz J, Shah BD, Jain MD, Balagurunathan Y, Locke FL. High metabolic tumor volume is associated with decreased efficacy of axicabtagene

© 2022 Butt OH et al. *JAMA Oncology.*

ciloleucel in large B-cell lymphoma. Blood Adv. 2020 Jul 28;4(14):3268-3276. doi: 10.1182/bloodadvances.2020001900. PMID: 32702097; PMCID: PMC7391155.
2. Sasanelli M, Meignan M, Haioun C, Berriolo-Riedinger A, Casasnovas RO, Biggi A, Gallamini A, Siegel BA, Cashen AF, Véra P, Tilly H, Versari A, Itti E. Pretherapy metabolic tumour volume is an independent predictor of outcome in patients with diffuse large B-cell lymphoma. Eur J Nucl Med Mol Imaging. 2014

© 2022 Butt OH et al. *JAMA Oncology.*

D. Supplemental Figures

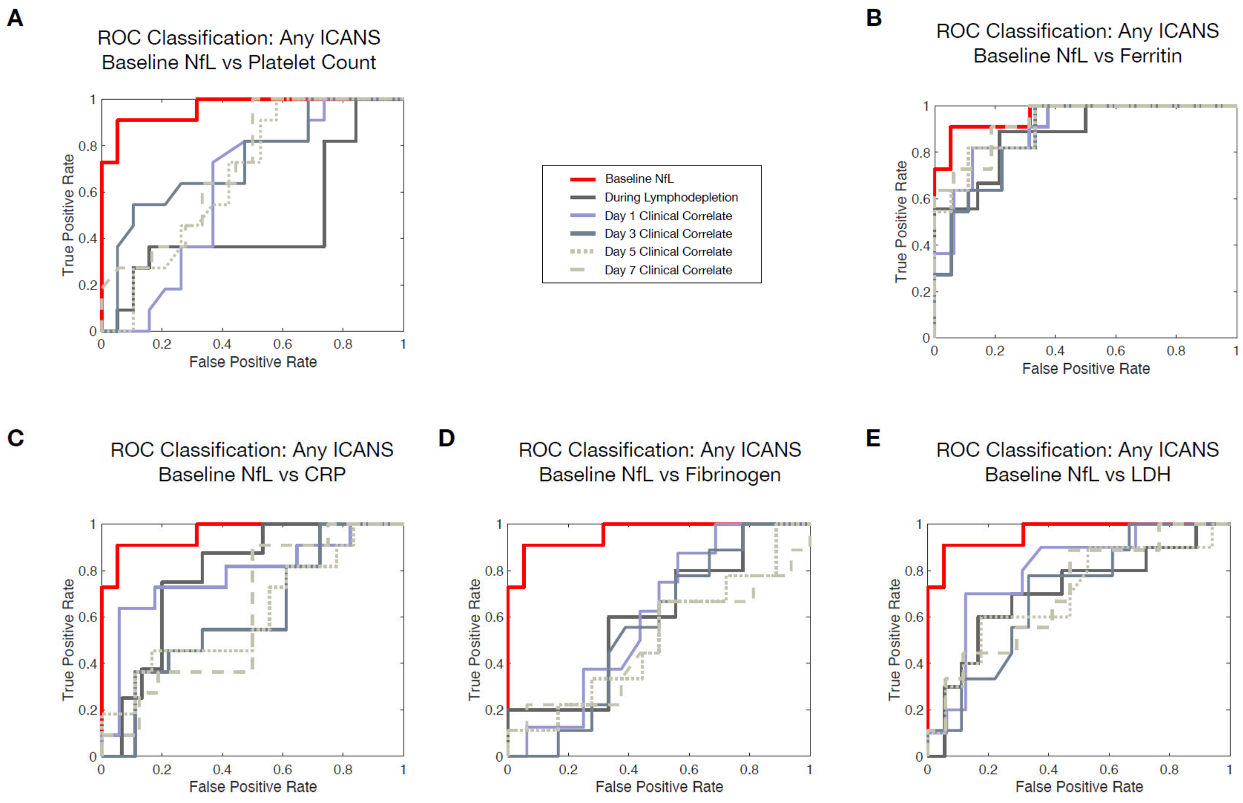

eFigure 1. Receiver operating characteristic curve classification of patients who develop any grade ICANS (1+) vs grade 0 for (A) Platelet Count, (B) Ferritin, (C) CRP, (D) Fibrinogen, (E) LDH. Five time-points are compared vs baseline NfL levels.

© 2022 Butt OH et al. *JAMA Oncology.*

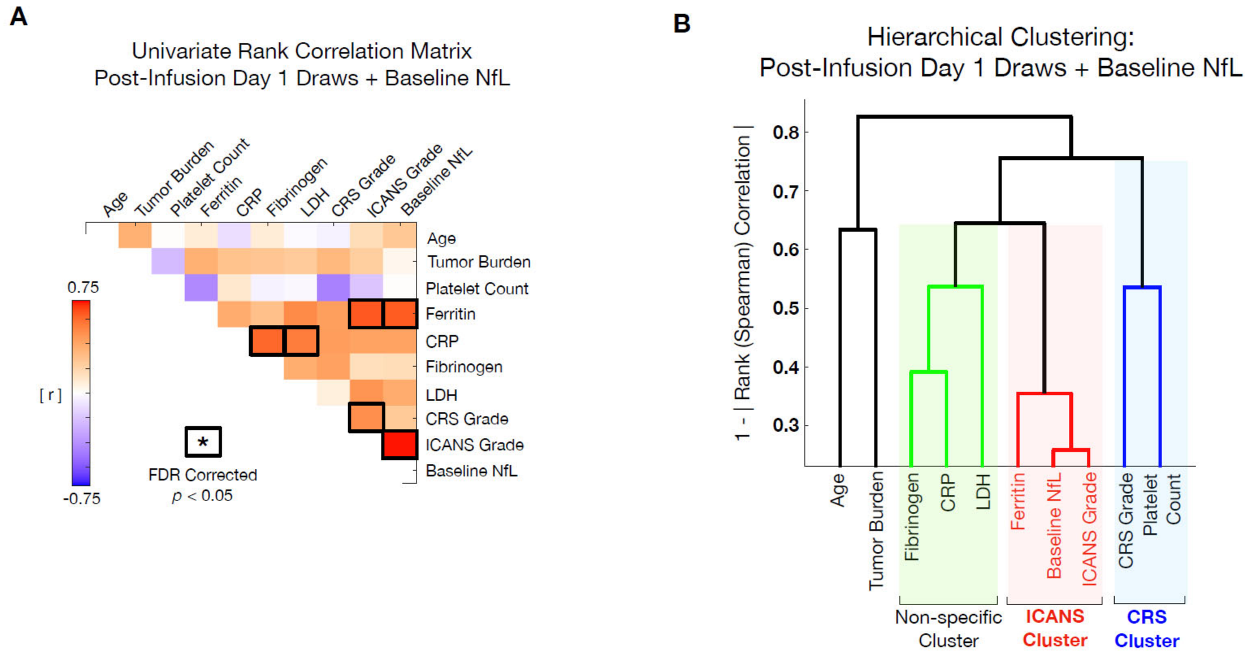

eFigure 2. (A) Univariate rank (Spearman's) cross-correlation matrix comparing **post-infusion day 1 (D1)** blood biomarkers to baseline NfL, age, and total tumor burden. Pairings which pass multiple comparison correction using false discovery rate (FDR) are outlined. (B) Hierarchical clustering of the same terms in (A). Clusters associating with ICANS labeled in red.

© 2022 Butt OH et al. *JAMA Oncology.*

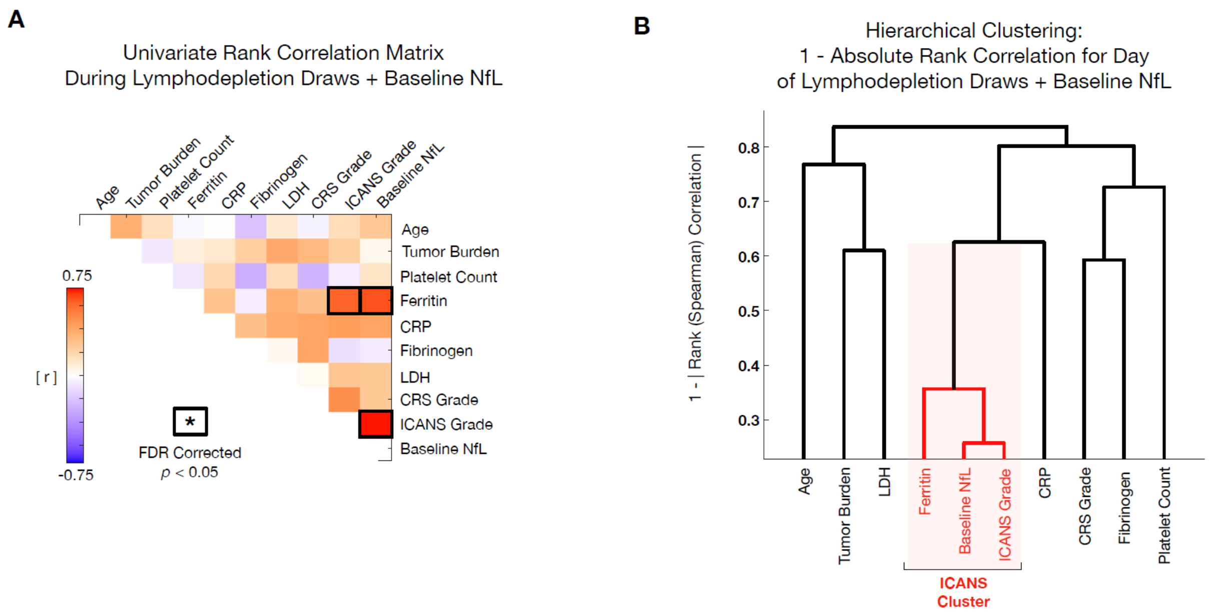

eFigure 3. (A) Univariate rank (Spearman's) cross-correlation matrix comparing **during lymphodepletion** blood biomarkers to baseline NfL, age, and total tumor burden. Pairings which pass multiple comparison correction using false discovery rate (FDR) are outlined. (B) Hierarchical clustering of the same terms in (A). Clusters associating with ICANS labeled in red.

© 2022 Butt OH et al. *JAMA Oncology.*

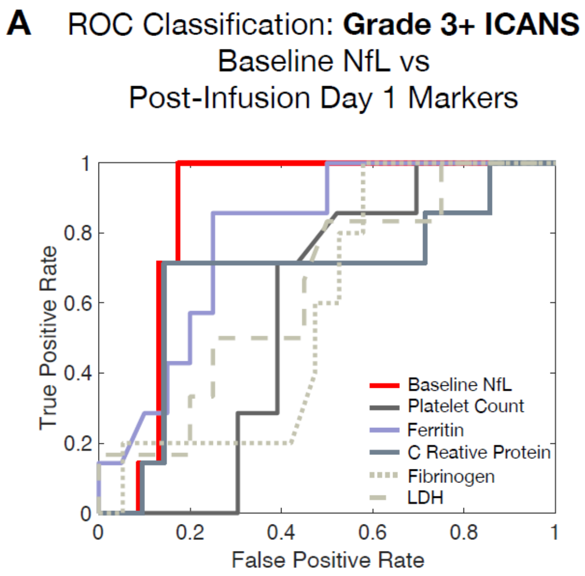

eFigure 4. Receiver operating characteristic curve classification of patients who develop grade 3+ ICANS vs grade 0-2 ICANS for baseline NfL and available post-infusion day 1 (D1) markers

© 2022 Butt OH et al. *JAMA Oncology.*

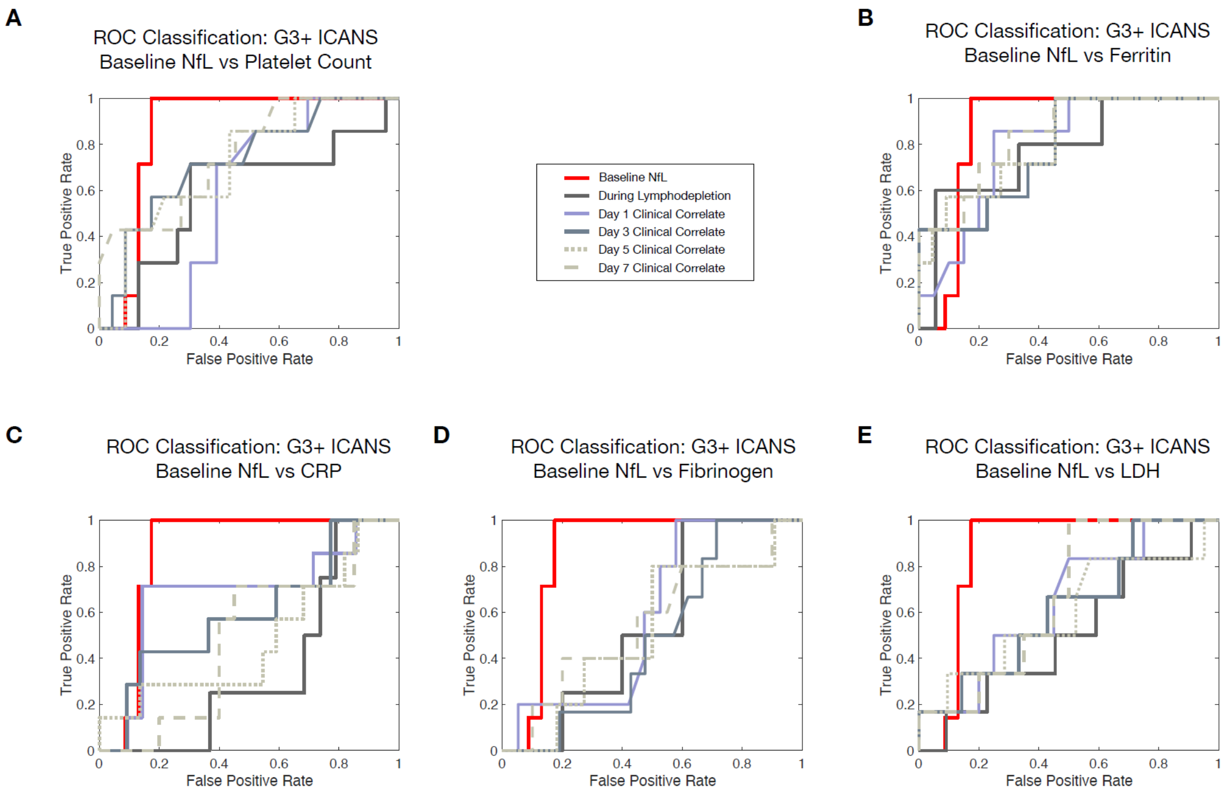

eFigure 5. Receiver operating characteristic curve classification of patients who develop grade 3+ ICANS vs grade 0-2 for (A) Platelet Count, (B) Ferritin, (C) CRP, (D) Fibrinogen, and (E) LDH. Five time-points (during lymphodepletion, post-infusion day 1 (D1), D3, D5, D7) are compared vs baseline NfL levels

© 2022 Butt OH et al. *JAMA Oncology.*

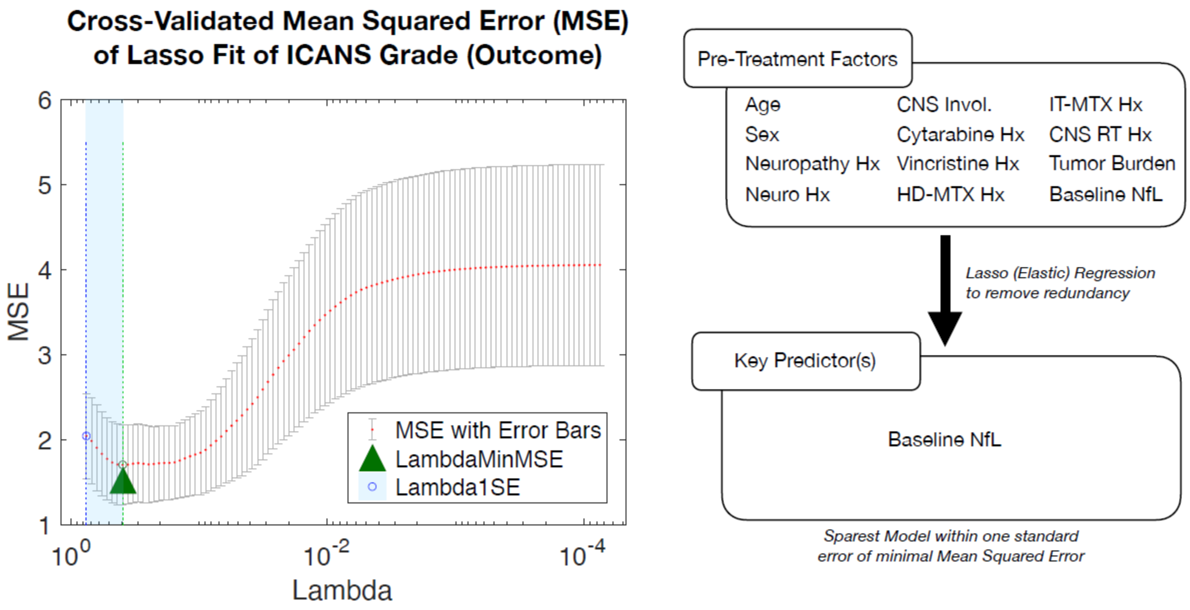

eFigure 6. Cross-Validation (12x) Mean Squared Error plot reflecting lasso (elastic) regression of 12 pre-treatment factors. A single predictor (log-normalized baseline NfL) is observed in the sparest model (green) within one standard deviation (blue) of the minimal mean squared error.

© 2022 Butt OH et al. *JAMA Oncology.*

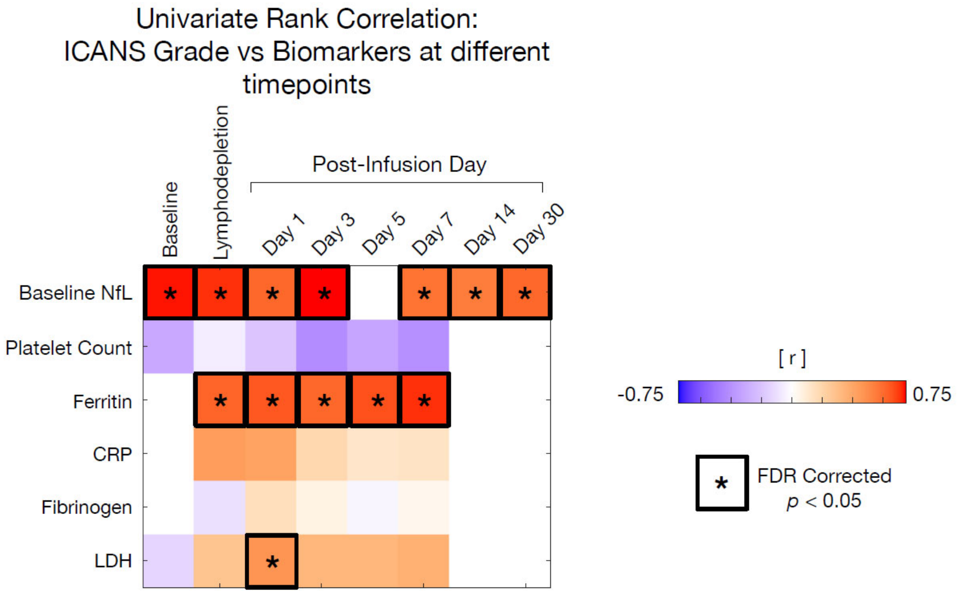

eFigure 7. Univariate correlation between ICANS grade and available biomarkers at different time-points relative to lymphodepletion and CAR T infusion. “Baseline” refers to prior to lymphodepletion, while “lymphodepletion” refers to blood draws obtained during lymphodepletion, but prior to CAR infusion. Significant relationships after correction for multiple comparisons using a FDR as outlined (*).

© 2022 Butt OH et al. *JAMA Oncology.*
